# Stokes on Diseases of the Heart

**Published:** 1855-12

**Authors:** 


### STOKES ON DISEASES OF THE HEART.

We are indebted to the publishers, Messrs. Lindsay & Blakiston, for a copy of this truly practical and excellent work. Our high estimate of its merits has already been expressed. No physician should be without it.

